# Uncharted territories: Solving the mysteries of male meiosis in flies

**DOI:** 10.1371/journal.pgen.1011185

**Published:** 2024-03-15

**Authors:** LingSze Lee, Leah F. Rosin

**Affiliations:** Unit on Chromosome Dynamics, Division of Developmental Biology, Eunice Kennedy Shriver National Institute of Child Health and Human Development, National Institutes of Health, Bethesda, Maryland, United States of America; College de France CNRS, FRANCE

## Abstract

The segregation of homologous chromosomes during meiosis typically requires tight end-to-end chromosome pairing. However, in *Drosophila* spermatogenesis, male flies segregate their chromosomes without classic synaptonemal complex formation and without recombination, instead compartmentalizing homologs into subnuclear domains known as chromosome territories (CTs). How homologs find each other in the nucleus and are separated into CTs has been one of the biggest riddles in chromosome biology. Here, we discuss our current understanding of pairing and CT formation in flies and review recent data on how homologs are linked and partitioned during meiosis in male flies.

## Introduction

Meiosis is the specialized cell division that generates haploid gametes in eukaryotic species. During the first meiotic division, homologous chromosomes (1 maternal and 1 paternal copy of the same chromosome) must segregate into separate daughter cells. Accurate chromosome segregation during meiosis I is facilitated by the juxtaposition of homologous partners, where the 2 chromosome copies find each other in 3D space and form associations to keep them connected until anaphase I. In most species, these associations form during a prolonged prophase I, which consists of chromatin decompaction and linearization to facilitate homolog recognition, alignment, and pairing/synapsis. Here, we distinguish between homolog alignment, which we define as the 2 homologous copies coming into close physical proximity in the nucleus and aligning from end-to-end, and pairing, which occurs after alignment and involves intimate inter-chromosomal associations that may or may not be dependent on the formation of the synaptonemal complex (SC). In conventional meiosis, the SC forms in a structurally conserved tripartite manner, with lateral elements along each homolog connected to central elements by transverse filaments (reviewed in [1,2]). These pairing interactions are stabilized by crossovers (COs) that form during genetic recombination, creating physical links between the homologs that remain even after dissociation of the SC known as chiasma (singular) or chiasmata (plural). The resulting bivalent structure is necessary for biorientation after nuclear envelope breakdown (NEBD) at metaphase I and accurate chromosome segregation at anaphase I (reviewed in [3]).

**Fig 1:**
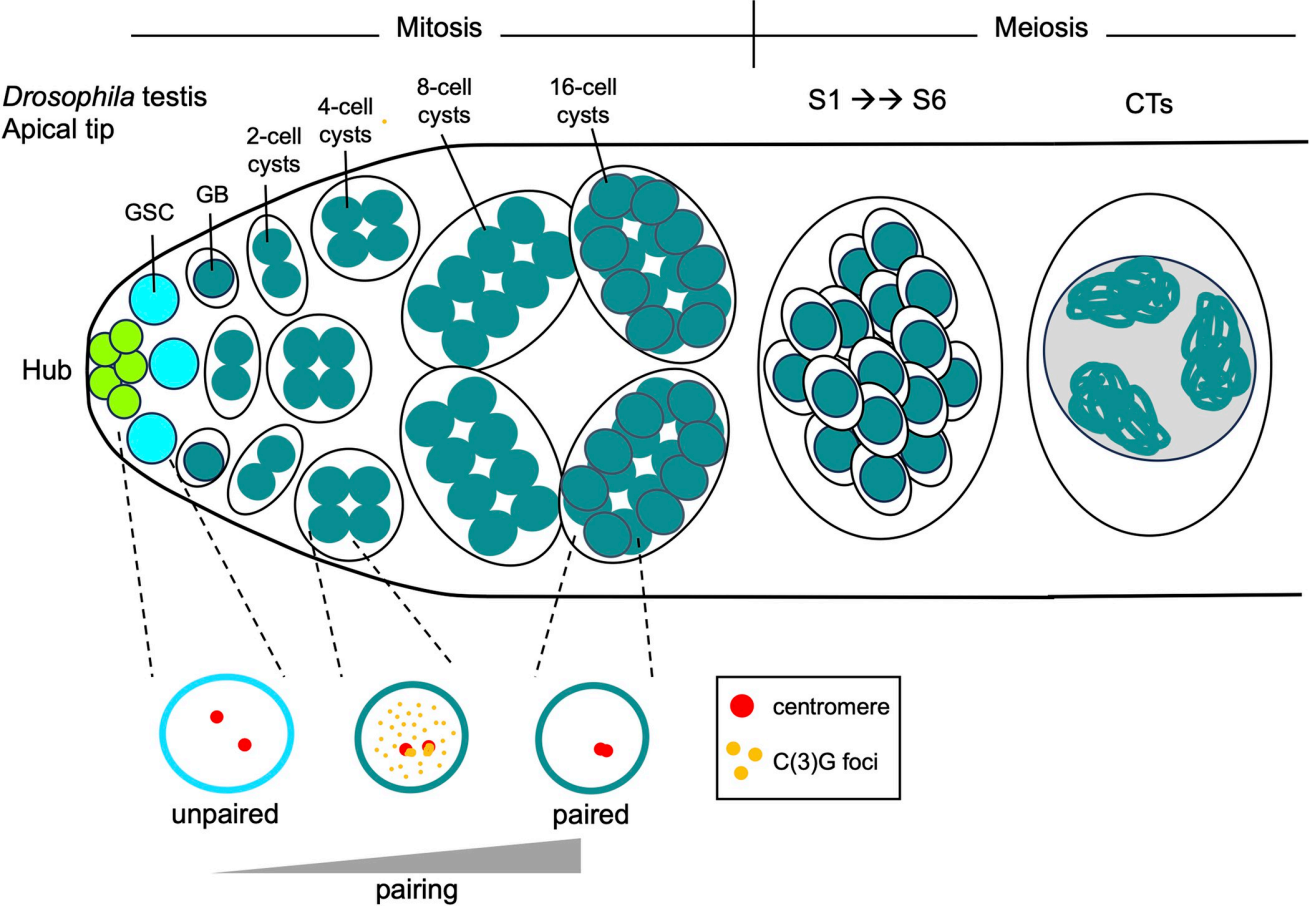
Cartoon schematic of germline progression in a *Drosophila* testis. *Top*, *from left to right*: somatic hub cells (bright green) are located at the most apical tip of the testis. These cells form a niche for the GSCs (cyan). GSCs undergo asymmetric mitotic divisions, producing a new daughter GSC and a GB (dark green). GBs undergo 4 rounds of mitosis with incomplete cytokinesis, resulting in the formation of 2-, 4-, 8-, and 16-cell interconnected spermatogonial cysts. The germ cells then undergo an extended G2/prophase I for cell growth, which can be divided into 7 substages: S1, S2a, S2b, and S3–S6. During this time, homologs are partitioned into separate regions in the nucleus known as CTs. The formation of CTs can be observed by only visualizing DNA. By the S2a stage, nuclei show a tri-lobular morphology, and by S2b, nuclei harbor 3 distinct chromatin masses. These 3 masses are the CTs containing the bivalents of the 3 major fly chromosomes (2, 3, and X/Y). The small fourth chromosome tends to share a territory with the sex chromosomes. By S5, the CTs in the mature spermatocyte nuclei are spread out, creating an approximately equilateral triangle. While only 1 cell with CTs is shown, all 16 cells from the cyst progress to this stage and have this chromosome morphology. *Bottom*: The pairing of homologs occurs progressively during the mitotic divisions. Two representative homologous centromeres are shown in red. C(3)G foci are shown in orange. In the GSCs, only 20%–30% of homologs are paired, but by the 16-cell cyst stage, more than 80% of homologs are paired. This pairing occurs concurrently with the formation of C(3)G foci, a transverse filament protein involved in *Drosophila* SC formation. C(3)G mutants fail to properly pair homologs by the 16-cell cyst stage, implicating the SC in proper homolog pairing in male *Drosophila* for the first time. CT, chromosome territory; GB, gonioblast; GSC, germline stem cell; SC, synaptonemal complex.

However, male *Drosophila* segregate their chromosomes in meiosis using a unique mechanism, without tripartite SC formation [4,5] and without recombination/chiasmata to link homologs together at the end of prophase I/metaphase I [6]. Instead, bivalents remain connected during spermatocyte maturation (Fig 1) via an alternative pathway, called alternative homolog conjunction (AHC; reviewed in [7]). Four AHC proteins have been identified so far: SNM (stromalin 2), MNM (modifier of mdg4), UNO (univalents only), and TEF (teflon) [8–10], but how and where the AHC proteins function during *Drosophila* male meiosis has remained elusive, and what prevents AHC proteins from linking non-homologous chromosomes together is unknown.

**Fig 2:**
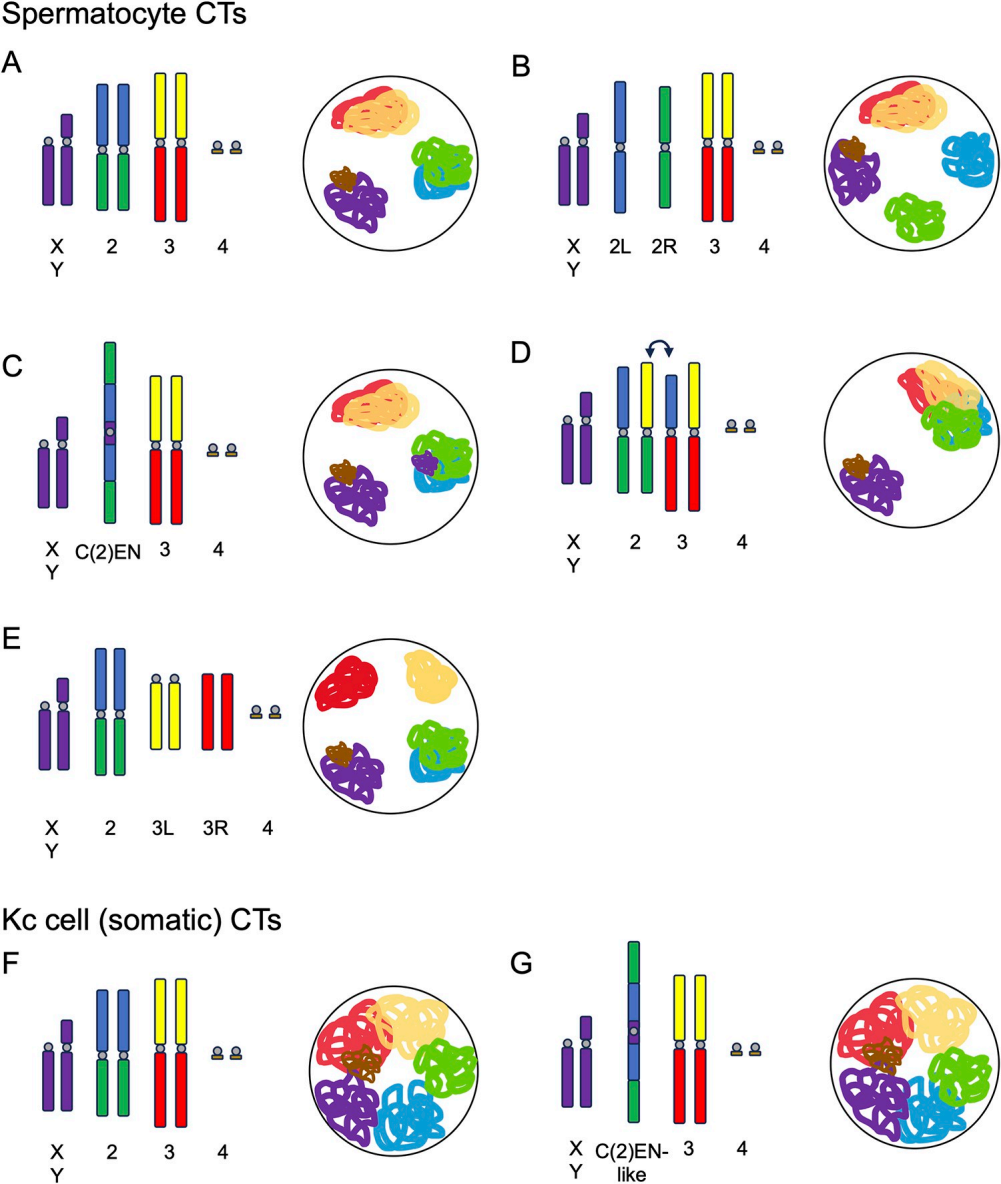
Karyotypic changes impact CT formation during spermatogenesis. Top: Karyotype and resulting CTs during fly spermatogenesis. *Drosophila* wild-type karyotype and CTs are shown in **A**. (**B–E**) Show various mutant karyotypes and the resulting CTs. G1 chromosomes (unreplicated chromatids) are shown for simplicity. Centromeres are shown in gray. A genome with 3 major chromosomes leads to the formation of 3 CTs (**A and C**). Karyotypes with 4 major chromosomes lead to the formation of 4 CTs (**B** and **E**). When inter-chromosomal translocations occur (for example, between chromosomes 2 and 3; **C**), this leads to the formation of quadrivalents and results in 2 major CTs. Bottom: Karyotype and resulting CTs formed in Kc (somatic) cells (**F** and **G**). In this context, homology almost entirely dictates CT formation, regardless of the underlying karyotypic changes. CT, chromosome territory.

Despite the lack of SC and chiasmata formation in fly spermatogenesis, homologs completely align and are sequestered into separate regions in the nucleus known as chromosome territories (CTs) [11]. The formation of CTs can be observed by only visualizing DNA. By mid-prophase I, nuclei show a tri-lobular morphology and harbor 3 distinct chromatin masses. These 3 masses are the CTs containing the bivalents of the 3 major fly chromosomes (2, 3, and X/Y; Figs 1 and 2A). The small fourth chromosome tends to share a territory with the sex chromosomes, although the mechanism behind this is unclear to this day. The CTs in the mature spermatocyte nuclei are spread out, creating an approximately equilateral triangle. Yet, how homologs are partitioned into their respective CTs and what prevents heterologous chromosomes from forming CTs together is unknown.

Here, we review recent studies elucidating the rules governing CT formation and chromosome pairing during *Drosophila* spermatogenesis.

### Pairing initiation in *Drosophila* male meiosis

How homologs find each other in *Drosophila* male meiosis is unclear, as is the case in most species. What has become clear in recent decades is that in most species, including fruit flies, nematodes, budding yeast, and mice, homolog alignment is independent of double-strand break (DSB) formation (reviewed in [12]). However, in most species, DSBs are required later in prophase I to stabilize pairing interactions. This is not the case for male *Drosophila*, which never form DSBs and never form COs, and yet homologs still associate [6]. Intriguingly, recent work from Rubin and colleagues investigating chromosome dynamics in the fly male germline suggests that meiotic pairing in male meiosis is not simply a continuation of pre-meiotic pairing in flies, as was previously believed to be the case. Instead, Rubin and colleagues show that homologs are unpaired in germline stem cells in the testes and that pairing of homologs is re-initiated during the mitotic division preceding meiosis (Fig 1) [13].

How do the homologs find each other? Studies from many species suggest that rapid chromosome movements facilitate the initial search for homology and homolog alignment. These movements are proposed to bring homologs closer in 3D space and also to help resolve entanglements between heterologous chromosomes (reviewed in [14]). Such movements have previously been shown to play a role in pairing in *Drosophila* females, where nuclear rotations and centromere movements are required to initiate pairing [15]. In their 2022 study, Rubin and colleagues demonstrate that movement also plays a role in *Drosophila* male meiosis, where slow chromosome movements occur during pairing initiation [13]. These movements are driven by microtubule motors connected to the *Drosophila* SUN/KASH protein complex, similar to meiotic chromosome movements in other species. Analysis of pre-meiotic pairing in mutants of the SUN-domain protein, Klaroid, and KASH-domain protein, Klarsicht, further demonstrated that these factors are required for pairing initiation in *Drosophila* males. Therefore, although pairing in male flies occurs during the pre-meiotic mitotic divisions, similar mechanisms seem to apply to the search for homology as in more canonical meiosis.

Despite previous beliefs that no SC forms in *Drosophila* male meiosis for linking homologs together [4,5,7], Rubin and team point out that according to both FlyAtlas2 and ModENCODE, the *Drosophila* SC proteins C(3)G and Corona are expressed in testes. In addition to validating this expression using RT-qPCR, Rubin and colleagues surprisingly found that C(3)G foci form along the chromosome arms by both IF and GFP-tagging during these early pairing events in the mitotic zone (Fig 1). This foci phenotype is highly reminiscent of insulator “buttons” that facilitate homolog pairing in fly somatic cells [16–18]. Additionally, studies using C(3)G and Corona mutants revealed that both these factors are required for the timely re-initiation of pairing in the mitotic region of the male germline, implicating SC proteins in pairing in *Drosophila* males for the first time. Still, despite widespread pairing defects in the mitotic zone of C(3)G and Corona mutants, flies remained fertile, suggesting that this SC-mediated pairing is likely redundant with other mechanisms regulating meiotic chromosome separation in *Drosophila* spermatogenesis.

Evidence for non-SC-mediated pairing in fly spermatogenesis comes from classical studies looking at X-Y pairing. While essentially no euchromatic homology exists between the X and Y sex chromosomes in flies, a combination of cytological and genetic studies by Kenneth Cooper and others as early as the 1960s revealed that a region of the shared rDNA repeats connects these chromosomes during spermatogenesis [19–21]. This thread-like rDNA connection, known as the collochore, keeps the sex chromosomes connected throughout prophase I and ensures their accurate separation at metaphase I. No evidence for such a genetic-based pairing mechanism has ever been validated for the autosomes in flies, and autosomal homolog recognition in flies remains largely elusive. However, pericentric heterochromatin and centromeres have been implicated in early pairing events in both male and female fly meiosis for all chromosomes [19–27], opening the door to the possibility that heterochromatin-associated factors could play a role in pairing initiation in this species.

### CT formation in *Drosophila* spermatocytes

Homolog pairing and CT partitioning are intimately linked in fly male meiosis. It is therefore unsurprising that the mechanism by which paired homologs are separated into their respective CTs was as elusive as pairing initiation itself. In one of their recent studies, Vernizzi and Lehner set out to determine how homologs, once paired, are sequestered to their separate CTs [28]. Previous work from Vernizzi and Lehner revealed that, like its role in the soma [29,30] and female germline [31], the Condensin II complex antagonizes inter-chromosomal interactions in the male germline and facilitates the individualization of chromosomal bivalents [32,33]. Once individualized, homologous associations are stabilized by the AHC complex [8–10].

Still, many questions remained, including how cells know which chromosomes to put into separate territories. Is each individual chromosomal entity (made up of linked homologs) partitioned into a separate CT? Or does chromosome identity factor into this mechanism? To test the model that territory organization is governed by forces maximally separating distinct chromosomal entities, Vernizzi and Lehner used a combination of live imaging and classical squash approaches to visualize CTs in testes from flies carrying a variety of chromosomal aberrations. In wild-type flies, 3 main territories are typically observed (for chr2, chr3, and chrX/Y/4). Interestingly, in flies carrying chr2 compound chromosomes, where each arm of chr2 forms a separate chromosome, Vernizzi and Lehner observed an additional, fourth territory (Fig 2B). Even with the addition of a fourth CT, all CTs were still evenly and maximally spaced out in the nucleus. The same phenomenon was observed for flies carrying chr3 compound chromosomes. As some heterochromatic repeats are likely shared between the arms of chromosomes, the formation of separate CTs by chr2L and chr2R compounds suggests that homology in heterochromatic domains does not prevent chromosomes from being partitioned into separate territories. Similarly, when the authors looked at spermatocytes from flies carrying a complex chr2 fusion which also contains repetitive elements typically found on the sex chromosomes, C(2)EN, they found that spermatocytes harbored 3 equally spaced CTs, similar to WT flies (Fig 2C). This observation suggests that (1) a lack of euchromatic homology between chr2L and 2R is not sufficient to drive chromosomes apart during CT formation; and (2) the presence of heterochromatic homology between the sex chromosomes and C(2)EN is not sufficient to bring chromosomes together into a single CT.

**Fig 3:**
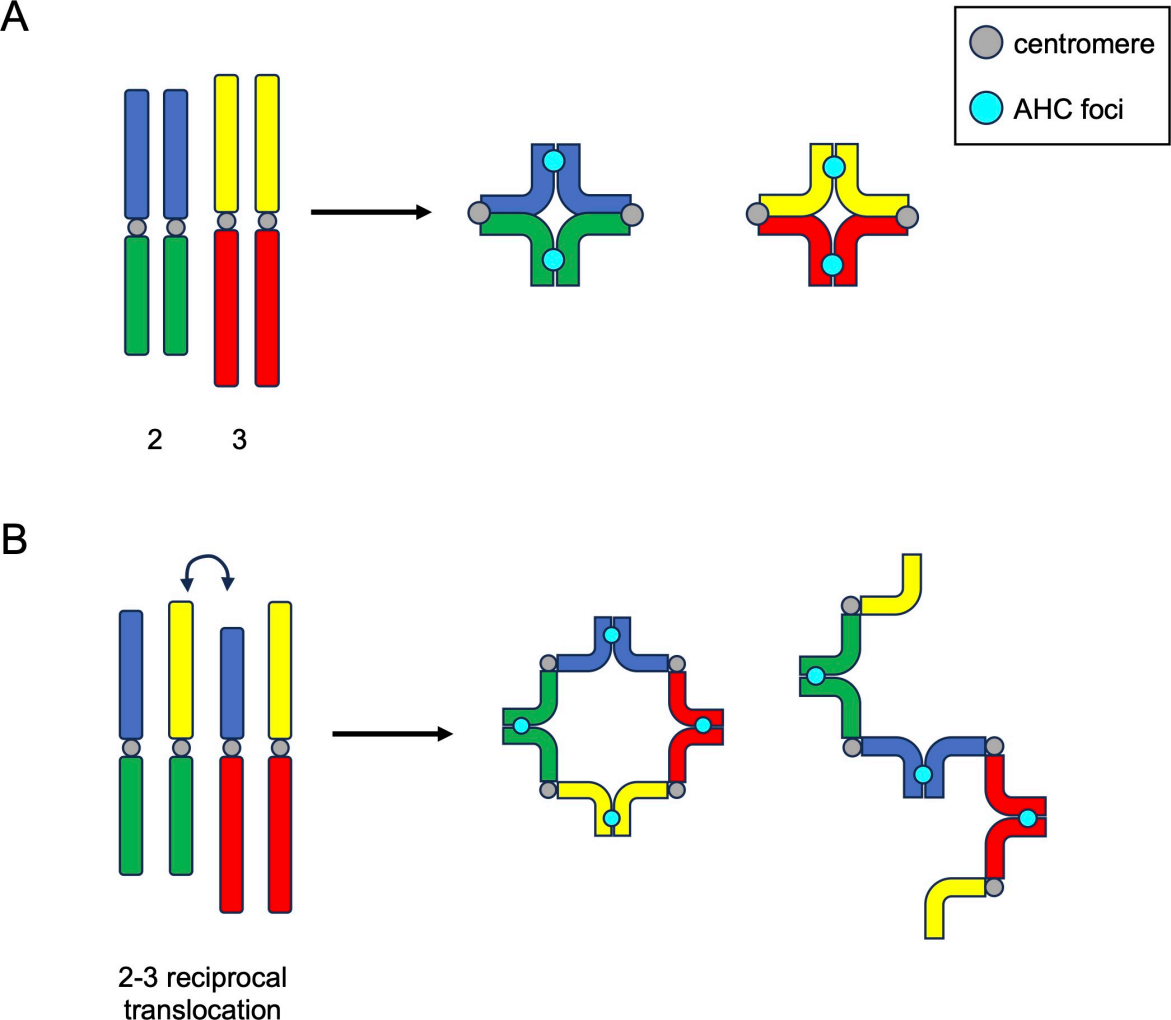
AHC foci link homologs in bivalents and quadrivalents in a mechanism akin to chiasmata formation. Left: *Drosophila* wild-type (**A**) or mutant (**B**) cartoon karyotypes. Centromeres are shown in gray. AHC foci are shown in cyan. (**B**) Shows a large translocation between chromosomes 2 and 3. G1 chromosomes are shown for simplicity. In wild-type cells, homologs form bivalent structures similar to those observed outside of *Drosophila*. However, in this instance, AHC foci link homologs in lieu of chiasmata. When quadrivalents form (B) either as rings (middle) or rods (right), AHC foci form equally spaced along the quadrivalent structure, in the same way canonical chiasmata would. AHC, alternative homolog conjunction.

Intriguingly, when CT formation was examined in flies heterozygous for reciprocal translocations between the 2 large fly chromosomes (chr2 and chr3), Vernizzi and Lehner observed only a single large CT (Fig 2D). One possible explanation for the formation of this single large CT is that all linked chromosomal units, regardless of identity, are segregated into the same CT, and all unlinked units form separate CTs. In this model, such reciprocal translocations would result in the formation of complex linkages between chr2 and chr3, forming a quadrivalent (4 linked chromosomes) during meiosis (Fig 3), which would lead to all chr2 and chr3 segments being partitioned into the same CT. Indeed, using live imaging, Vernizzi and Lehner showed that spermatocytes from flies with chr2-chr3 reciprocal translocations harbor either large ring-, square-, or rod-shaped quadrivalents (Fig 3). Importantly, the ring-shaped quadrivalents observed in spermatocytes with reciprocal translocations between chr2 and chr3 highly resemble quadrivalent rings that form in canonical meiosis with reciprocal translocations, for example, in mice [34] and plants [35]. The fact that quadrivalents between heterologous chromosomes form a single CT further supports the idea that chromosomal identity does not play a role in CT formation in the male germline, and instead, linkages between chromosomes during CT formation dictate how chromosomes are partitioned into CTs during spermatogenesis in flies.

In an even more striking experiment to support this notion, Vernizzi and Lehner then used CRISPR technology to cut chr3 in half by targeting Cas9 to the *dodeca* pericentric satellite repeat on chr3. This separation of the left and right halves of chr3 led to 4 CTs in spermatocytes (Fig 2E). Most interestingly, one of these 2 chr3 sub-CTs almost always lacked a centromere (as expected for the position of *dodeca*), and yet both sub-CTs were almost always able to retain the maximized spacing in the nucleus. This result corroborates the model that individualized, unlinked chromosomal entities form their own CTs and further suggests that centromere-associations with the nuclear envelope are not involved in the spatial separation of CTs.

This paradigm where individualized entities form separate CTs regardless of chromosomal identity is in contrast to findings of CT formation in fly somatic cells. In 2018, Rosin and colleagues showed that chromosome identity almost entirely dictates CT partitioning in the soma [29]. *Drosophila* cultured cell lines harboring heterologous translocations still form normal interphase CTs by Oligopaints FISH. For example, a translocation between chr2 and chr3, similar to C(2)EN, does not disrupt the chr2 or chr3 territories, and each piece of DNA joins its endogenous CT (Fig 2F and 2G). However, these types of translocations do alter the position of interphase CTs, bringing the translocated chromosomes closer together in interphase [29].

Together, these results suggest that chromosomal identity (i.e., being part of chr2) is not sufficient to group chromosomes together into the same territory and does not prevent chromosomes from moving apart and forming separate CTs in spermatocytes. Instead, these data all support a model where individualized chromosomes are all separated into distinct CTs which are then maximally spaced out in the nucleus.

### Links between homologs and the AHC

In some species, links between homologs during meiosis are achieved in a crossover-independent manner. For example, females of the silkworm moth *Bombyx mori*, males of *Drosophila*, and the nematode *C*. *elegans* all form inter-chromosomal linkages independent of crossovers [36–40]. *Drosophila* males, specifically, employ a completely alternative mechanism to pair homologs in meiosis called “alternative homolog conjunction” (AHC). While it has been suggested that AHC assembly occurs at the same time or slightly after CT formation [41], which would prevent non-homologous associations, how the 4 AHC proteins (TEF, SNM, MNM, and UNO) interact with each other and chromosomes to link homologs together was not clear.

In 2 recent studies, Kabakci and colleagues investigated how AHC proteins assemble and bind to DNA [42,43]. These studies had several key findings. First, TEF binds directly to chromosomes and recruits MNM in early meiosis, when AHC is first established. However, TEF is no longer present in late spermatocytes [9,43], suggesting that TEF does not play a major role in maintaining homolog linkages later in meiosis. Second, SNM, UNO, and MNM (collectively known as “SUM”) remain localized to bivalents throughout early meiosis, initially forming weak foci along bivalents and in the sub-nucleolar region of the nucleus [8–10]. Interestingly, these weak SUM foci begin to coalesce into 1 single, bright focus randomly placed along chromosomal bivalents around the same time as NEBD and chromosome condensation during meiosis I (Fig 3; [28,43]).

This change in the number and structure of AHC foci is remarkably similar to protein diffusion and “coarsening” observed recently for pro-CO factors in plants and nematodes [44–48]. Accurate homolog segregation in meiosis I in these and many other species is ensured by the formation of COs that link homologs together until anaphase I. CO formation is tightly regulated: there needs to be at least 1 CO per chromosome pair, but not more than a few COs, and the COs that do form need to be spatially separated along the chromosome axis (CO assurance and CO interference, respectively; reviewed in [49,50]). Pro-CO factors initially localize throughout the SC, forming multiple small foci termed early recombination nodules (RNs), but later become restricted to a single, or at most a few, large foci (late RNs, [51]). Recent studies in both plants and nematodes have suggested that this change in RN number and structure occurs through a phase-separation-based mechanism where pro-CO factors act as biomolecular condensates that “coarsen” and aggregate throughout prophase I [45–48]. The changes in the structure and number of RNs that occur as a result of “coarsening” are highly similar to the changes in the structure and number of AHC foci observed by Lehner and colleagues, supporting a model where AHC foci are functionally replacing crossovers in fly male meiosis.

Further support for this model comes from the ring-shaped quadrivalents observed in the same studies. Along the ring-shaped quadrivalents that formed in spermatocytes with reciprocal translocations between chr2 and chr3, “mature” AHC foci are evenly spaced within the quadrivalent, similar to CO position in quadrivalents in canonical meiosis as a result of CO interference (Fig 3; [28]). Intriguingly, such a “coarsening” of homolog-connecting factors is likely not limited to RNs and AHC foci. A recent study in female *Bombyx mori*, which also employ an achiasmatic meiosis, demonstrated that SC components remain between homologs throughout prophase I but become dramatically restructured, gradually becoming dense proteinaceous globs between homologs by metaphase I [52,53]. This could also presumably occur by a mechanism akin to the coarsening of biomolecular condensates, leading us to speculate that “coarsening” could be a universal aspect of inter-homolog associations.

Finally, Kabakci and colleagues used a spectrum of computational and biochemical assays to show that SUM proteins SNM and UNO are Cohesin-related proteins that facilitate homolog linkages. UNO is an α-kleisin-like protein analogous to Rad21 with a maintained Separase cleavage site [10]. Interestingly, the conjunction made by SUM proteins does not seem to involve DNA entrapment, as it does with canonical Cohesin, but rather direct DNA binding of SUM multimers in a sequence-nonspecific manner [42].

In summary, these results reveal the similarities between AHC-based homolog linkages and chiasmata in canonical meiosis.

### Conclusions and summary

How homologs find each other in 3D space and pair from end-to-end remains one of the biggest unsolved mysteries in chromosome biology. In this review, we recap recent findings regarding the DSB- and CO-independent pairing events that occur in *Drosophila* spermatogenesis. While homolog pairing is nearly ubiquitous in fly somatic cells [54], germline pairing in both females [15] and males [13] is not simply a continuation of somatic pairing. In males specifically, pairing is re-initiated during the pre-meiotic mitotic divisions in germ cells. This pairing involves chromosome movements directed by microtubules, as well as the SC proteins C(3)G and Corona [13]. Once paired, homologs are partitioned into spatially separated CTs. This process is at least partially driven by the action of Condensin II, which antagonized inter-chromosomal interactions to help separate bivalents [30–33]. The recent studies discussed here further reveal that this process sequesters any linked chromosomal units into a single CT, regardless of chromosome identity [28]. After CT formation, homolog–homolog interactions are stabilized by the AHC pathway [8–10]. These recent studies demonstrate that AHC SUM proteins are Cohesin-related proteins that multimerize, bind directly to DNA in a sequence-independent manner, and are cleaved by Separase for homolog separation [42,43]. Importantly, SUM proteins clearly link homologs in a mechanism similar to canonical COs. SUM proteins coalesce into distinct foci along chromosomes which avoid pericentromeric regions and form around one focus per chromosome, and these foci are equidistant on complex chromosomal structures like quadrivalents [28]. These new discoveries have elucidated many of the perplexities of the recombination-independent mechanisms occurring in fly spermatogenesis. Further studies will be required to validate the similarities between canonical CO formation and the fly AHC pathway and to clarify exactly how AHC proteins are removed after metaphase I.
